# Practical Notes and Formulæ

**Published:** 1883-04-20

**Authors:** 


### PRACTICAL NOTES AND FORMULÆ.

**Anti-Diphtheritic Inhalations.**—Some years ago, Dr. H. Hager recommended a mixture composed of—

| | |
|---|---|
| Carbolic acid | 10 parts, |
| Alcohol | 10 " |
| Water of ammonia | 12 " |
| Distilled water | 20 " |

As an excellent inhalation in catarrhal affections. It was directed to be used thus: A small wide-mouthed bottle was to be filled one-third with the liquid; then a sufficient quantity of cotton was to be introduced to just soak up all the liquid. The bottle was then to be well stopped. In coryza, incipient catarrh, or similar affections, the inhalation through the nostrils of some of the vapor of the compound was found to be of the greatest benefit.

The same author now recommends a still stronger compound, to be made from

| | |
|---|---|
| Carbolic acid | 10 parts, |
| Oil of turpentine—(or oil of eucalyptus) | 5 " |
| Water of ammonia | 12 " |
| Alcohol | 20 " |

A small quantity of this is to be dropped into a small wide-mouthed bottle half filled with cotton or asbestos, and the bottle well stopped. After a few days a little more may be added, until a strong odor is given off, when the bottle is opened.

A physician to whom Dr. Hager recommended the use of the compound thinks that it prevents the spread of diphtheria, since in five families, in each of which one case of diphtheria had become developed, its further spread was arrested, apparently through the use of the antiseptic inhalation.—*Med. News.*

**Gonorrhœa.**—Dr. Logan, of Florida, in Eclectic Medical Journal, says: The treatment I have had the most success with and that which seems to be nearer a specific than any other, is as follows:

| | |
|---|---|
| ℞ Calamine | grs. lxxx, |
| Powdered kino | grs. xxx, |
| Sulph. zinc | grs. x, |
| Sulph. morphine | grs. viij, |
| Boiling water | Oj, |

M. Sig. Shake and inject a syringeful every two hours, urinating each time before injecting. The injection should be retained two full minutes, then allowed to escape slowly, so as to leave the sediment in the urethra. The kino must be pulverized and dusted through a fine cloth so as to free it from lumps.

Out of 18 cases treated with the above, there was not a single case in which a cure was not effected inside of 14 days after commencing treetment. Cases seen early in the attack yielded in half that time.

**In Anorexia.**—M. Huchard recommends the following stomachic for persons who suffer from debility, with loss of appetite—

| | |
|---|---|
| Tinct. cardamoni | f ʒ i, |
| Tinct. anisi | f ʒ i, |
| Tinct. aurantii corticis | aa f ℥ iiss |
| Tinct. gentianæ | |
| Aquæ menth. pip | |
| Aquæ | ad ℥ ii. |

M. Sig. Teaspoonful between meals.—*Journal of Chem.*

**Elixir of Salicylic Acid.**—Dr. Wolff, according to the Journal of Pharmacy, gives the following formula—

Dissolve salicylic acid, ʒi. in alcohol, f. ʒvi., and add simple elixir (or elixir curacoa) q. s., f. ℥vi.

The dose is a tablespoonful, containing five grains of salicylic acid, the taste of which is well masked. The elixir should not be given with water. The additional amount of alcohol in this preparation is not contraindicated, but seems to overcome the tendency of the salicylic acid to act as a cardiac depressor.—*Ex.*

**Cosmetic Preparations.**—The following recipes are from good authority—

TAN AND FRECKLE LOTION.

| | |
|---|---|
| Corrosive sublimate | gr. vi, |
| Dilute hydrochloric acid | ʒ i, |
| Water | ℥ iv, |
| Alcohol | each ℥ ij, |
| Rose water | |
| Glycerine | ℥ i. |

Apply at night and wash from the skin with soap in the morning.

LE BON'S PERFUMED CARBOLIC ACID.

| | |
|---|---|
| Carbolic acid | 1 part, |
| Oil of lemon | 3 parts, |
| Alcohol (30°) | 100 parts. |

GLYCEROLE OF ARNICA.

| | |
|---|---|
| Fluid extract arnica | 10 parts, |
| Glycerine | 10 parts. |

For chapped hands insect bites, etc.

FRENCH COLD CREAM.

| | |
|---|---|
| Quince mucilage | 40 parts, |
| Almond soap | 1 part, |
| Stearic acid | 10 parts, |
| Glycerine | 2 parts. |

—*Journal of Chemistry.*

**A Cholagogue Formula.**—Dr. Blackwood publishes in the Medical Times, Philadelphia, the following fomula, which he has found very servicable in many a stubborn case of dyspepsia that had run the gauntlet unavailingly of all sorts of peptonoids. It is also, he says, an admirable cholagogue on general principles. It is certainly correctly based on the physiological action of its base and adjuvants as demonstrated by the experiments of Rutherford:

| | | |
|---|---|---|
| ℞ | Cinchonidiæ sulphatis | aa ʒ ss, |
| | Euonymin | |
| | Leptandrin | |
| | Iridin | |
| | Juglandin | |
| | Podophyllin | aa gr. x. |
| | Ext. belladonna | |
| | Ext. nucis vomicæ | |
| | Ext. hyoscyami | |

M. ft. mass. et. div. in pil. no. lx. Sig. One or two at bed time. *Med. Age.*

**Turpentine for Tænia Solium.**—In the Southern Clinic, Dr. H. L. Harris relates the case of a mulatto girl to whom he gave on a Thursday afternoon—

| | | |
|---|---|---|
| ℞ | Ol. terebinthinæ | ℥ ss, |
| | Ol. ricini | ℥ iss. |

Sig. Tablespoonful at a dose.

And directed that one dose be taken, and that the patient retire fasting, and await developmets until one o'clock Friday.

At 12:20 o'clock Friday, he was summoned and found that she had passed a monster tape-worm, measuring seventeen and a half feet in length, careful measurement. She suffered from soreness over the hepatic region, but the abdomen has fallen, and the patient feels well.—*Med. and Surg. Rep.*

**New Compound Cathartic Pill.**—Dr. Palmer, of Florida, in Journal of Pharmacy, recommends the following as a substitute for the officinal compound cathartic pill, on account of its being less drastic, and more cholagogue in their action. It has been thoroughly tested in this part of the country, and is found superior to the United States formula in every particular, and I respectfully suggest it for consideration by the revisers of our standard. In this the calomel is increased and the gamboge diminished, as follows:

| | | |
|---|---|---|
| ℞ | Ext. coloc. comp | aa gr. 1⅓ |
| | Calomel | |
| | Ext. jalap | gr. 1, |
| | Gamboge | gr. 1-5. |

